# Cytotoxic activity of glycoalkaloids extract from fruits of *Solanum lycocarpum *A. St.-Hil

**DOI:** 10.1186/1753-6561-8-S4-P7

**Published:** 2014-10-01

**Authors:** Flávia Barbosa, Mariza Miranda, Walace Rizo, Bianca Bertoni, Jairo Bastos, Mozart Marins, Ana Fachin

**Affiliations:** 1 Unidade de Biotecnologia, Universidade de Ribeirão Preto (UNAERP), Ribeirão Preto, Brazil; 2 Laboratório de Farmacognosia, Faculdade de Ciências Farmacêuticas de Ribeirão Preto, University of São Paulo (USP-FCFRP), Brazil

## Background

Phytochemical analysis of the fruits of *Solanum lycocarpum*, popularly known as "fruit of the wolf", showed high concentrations of steroidal alkaloids, such as solasonine and solamargine which are substances with potential cytotoxic activity [1]. The cytotoxic activity displayed by these compounds may be due to presence of sugar moieties (rhamnose). Since there is interaction of glycoalkaloids with plasma membrane cholesterol leading to loss of membrane integrity, resulting in alterations in its permeability what induces cell death [2]. The aim of this study was to evaluate the cytotoxicity activity of the glycoalkaloids solamargine and solasonine purified from *S. lycocarpum *and commercial α-solanine alkaloid from potato sprout toward cell lines MCF-7 (human breast adenocarcinoma cell line), B16 (murine skin) and 3T3 (normal mouse embryo fibroblasts) by MTT.

## Methods

The cells lines were cultured at 37°C in a humidified atmosphere containing 5% of CO2 supplemented with 15% fetal bovine serum, using Dulbecco's modified Eagle's medium (Sigma). Penicillin (100 U/mL), streptomycin (100 ug/mL) and ciprofloxacin (100 ug/mL) were added to the medium to prevent bacterial growth. A stock solution (10 mg/mL) of glicoalkaloid was prepared in 5% DMSO. Solamargine, Solasonine purified from *S. lycocarpum *and alfa-Solanine (Sigma) was directly diluted in the medium to obtain concentrations ranging from 57.5 to 5.75 uM. The final concentration of DMSO was less than 0.5% and it had no negative effects on the cell lines. Cells were trypsinized (0.15% trypsin and 0.02% EDTA), counted in a hemocytometer (2.5x105cells/well), and incubated in a 96-well plate for 24 h. After addition of the alkaloid or vehicle dissolved in fresh medium, the cells were cultured at 37 °C in a 5% CO2 atmosphere for 48 h, and cytotoxicity was analyzed by the MTT assay. For this purpose, 20 uL MTT/well (5 mg/mL in Hanks solution) were added to the 96-well plate, and the assay was incubated for 4 h under the same conditions. Hereafter, the plates were measured through 550nm wavelength analysis, using an ELISA reader. Treatments were compared to negative control (medium with 0.5% DMSO) and positive controls Doxorubicin (0,258uM) and actinomycin D (0,119uM) [3]. Cytotoxicity was calculated by the formula: percent cytotoxicity = (1-[absorbance of experimental wells/absorbance of control wells])×100%. IC50 values were also determined. Data were analyzed by the Sisvar software.

## Results and conclusion

For the cell line MCF-7 the IC50 of Solamargine, solasonina and α-solanine were 13.55, 14.57 uM and 51.23 uM, respectively. The 3T3 cell line showed IC50 value of 20.11 uM, 13.47 and 49.79 of Solamargine, solasonina and α-solanine, respectively. Tested compounds (solamargine, solasonina and α-solanine) showed higher cytotoxicity to B16 with IC50 values of 34.075, 22.611 and 57.380 uM, respectively. Finally the tested glycoalkaloids showed pronunciated cytotoxity activity and may be further explored for the development of potential lead compounds active against cancer cells.

## References

[B1] Nakamura S Hongo M Sugimoto S Matsuda H Yoshikawa M Steroidal saponins and pseudoalkaloid oligoglycoside from Brazilian natural medicine, ''fruta do lobo" (fruit of Solanum lycocarpum) Phytochemistry 2008 69 1565 1572 10.1016/j.phytochem.2008.02.003 18353405

[B2] Vieira PM Da Costa PM Silva CRS Chen-Chen L Assessment of the Genotoxic, Antigenotoxic, and cytotoxic activities of the ethanolic fruit extract of Solanum lycocarpum A. St. Hill. (Solanaceae) by Micronucleus Test in Mice Journal of Medicinal Food 2010 13 6 1409 1414 doi:10.1089/jmf.2009.0295 10.1089/jmf.2009.0295 21091254

[B3] Rizo WF Ferreira LE Colnaghi V Martins JS Franchi LP Takahashi CS Beleboni RO Marins M Pereira PS Fachin AL Cytotoxicity and genotoxicity of coronaridine from Tabernaemontana catharinensis A.DC in a human laryngeal epithelial carcinoma cell line (Hep-2) Genet Mol Biol 2013 36 1 105 110 doi: 10.1590/S1415-47572013005000010 10.1590/S1415-47572013005000010 23569415 PMC3615513

